# Cognitive training via mobile app for addressing eating disorders’ cognitions in adolescents: a randomized control trial protocol

**DOI:** 10.1186/s40359-024-01772-z

**Published:** 2024-05-14

**Authors:** Marta Corberán, Sandra Arnáez, Yuliya Saman, Belén Pascual-Vera, Gemma García-Soriano, María Roncero

**Affiliations:** 1 Departamento de Personalidad, Evaluación y Tratamientos Psicológicos. Facultad de Psicología y Logopedia, Universitat de València, Av. Blasco Ibañez, 21, Valencia, 46010 Spain; 2 Departamento Departamento de Personalidad, Evaluación y Tratamientos Psicológicos, Facultad de Psicología, UNED, C/Juan Rosal, 10, Madrid, 28040 Spain

**Keywords:** Eating disorders, Adolescence, Mobile application, Dysfunctional beliefs

## Abstract

During adolescence, individuals are particularly vulnerable to developing eating disorders (EDs). To address the dysfunctional beliefs linked to these disorders, a new mobile app has been developed. This app, called GG eating disorders-Adolescents (GGED-AD), was created based on CBT to help adolescents work on their self-dialogue related to the core beliefs of eating disorders. The objective is to present the protocol for a randomized controlled trial to explore the efficacy of GGED-AD.

**Methods **The study will be carried out in adolescents aged 13 to 16 from an educational center in the Valencian Community. The participants will be randomized into two groups: the experimental group will use the GGED-AD app during 14 consecutive days for approximately 5 min each day; and the control group will use a neutral app (GGNEUTRAL) during the same time. Both groups will complete instruments that assess dysfunctional beliefs related to eating disorders, eating symptoms, symptoms of depression and anxiety, body satisfaction and self-esteem before and after the intervention. A follow-up will be conducted one month later.

**Results** A decrease in the degree of ascription to dysfunctional beliefs associated with eating disorders and eating symptomatology is expected, as well as an increase in body satisfaction and the self-esteem of the participants of the experimental group.

**Discussion **The app in this study could help tackle and prevent ED-related symptoms in adolescents.

**Trial registration** NCT06039514.

**Supplementary Information:**

The online version contains supplementary material available at 10.1186/s40359-024-01772-z.

## Background

Eating disorders (EDs) are complex mental health conditions characterized by a pathological pattern of preoccupation with weight and body shape control, inappropriate food intake, fear of gaining weight, and body image dissatisfaction [1]. While eating disorders can affect individuals of all ages, they commonly emerge during adolescence, a critical period of growth, development, and identity formation. Currently, the prevalence of adolescents with a diagnosis of ED ranges from 0.3% to 1.7% [2–4]. Due to their severity, comorbidity, and mortality risk, these disorders have serious repercussions on the physical and psychological development of children and adolescents [5, 6]. In addition, a worsening of mental health among young people has been observed as a consequence of the COVID-19 health pandemic and home confinement. This has led to an increase in the prevalence of EDs [7], as well as an aggravation of symptomatology in those already suffering from it [8, 9].

Cognitive behavioural therapy (CBT) is the most empirically supported psychological treatment for eating disorders [10, 11]. However, accessing treatment is difficult, and less than 20% of patients in need actually receive it [12]. In the adolescent population, this lack of treatment is even more noticeable [13]. In addition, its efficacy is not entirely satisfactory, as patients exhibit difficulties when it comes to recovery, such as a tendency toward chronicity, resistance to change, high level of comorbidity, and a significant percentage of relapses [14, 15]. All this reflects the existing need to work on the risk factors of eating disorders in order to minimize their occurrence.

In order to improve the prevention and treatment of EDs, the use of information and communication technologies (ICTs) has been developed in recent years. Regarding ICTs in EDs, these have been implemented in their assessment, prevention, treatment, and follow-up in various formats such as: virtual reality [16, 17], CBT via videoconferencing [18, 19], or on-line familiar therapy [20]. In terms of prevention programs, various projects have been implemented among adolescents and young people in schools. For example, the program *StudentBodies* conducts CBT online sessions in which a facilitator or guide creates group discussions about weight concerns. It consists of 8 modules of 30 min each. Different studies on its efficacy in a sample at risk of developing ED showed no significant reduction in the onset of future eating disorders [21–23]. Another example is the *eBodyProject* program. It is based on *BodyProject*, which is a program of 3 or 4 sessions guided by a clinician in which group exercises are carried out to explore the negative effects of following a thin body ideal [24]. When the program was conducted online, the acceptability was found to be better than when it was done in person [25] and studies indicate that the virtual delivery of the Body Project significantly decreased the likelihood of developing eating disorders in the future [26].

However, despite the existence of these prevention programs, there are none that are based on a mobile app. These tools have many advantages, such as accessibility anytime and anywhere, low cost, wide reach, reduced hesitation to seek assistance, and anonymity [27]. They do not require any clinician or other guide and, furthermore, young people are familiar with its use.

Regarding mobile applications for ED, different apps can be found in English, such as *Recovery Record,* focused on the self-management of typical ED behaviors and related thoughts and feelings and includes therapist feedback. Studies analyzing *Recovery Record*’s efficacy in the clinical population show that the experimental group did not obtain better results in the reduction of ED symptoms compared to the control group [28, 29]. However, the application was well received, and improved its users’ adherence and referral rates [30–32]. There is another app, called *Noom Monitor,* which is designed for self-monitoring meals, compensatory behaviors, exercise, body checking, cravings, and weight. The use of this app together with regular CBT revealed better results in two studies with adult patients with binge eating disorder and bulimia nervosa compared to patients who only received regular CBT [33, 34]. The app named *Break Binge Eating,* is based on the principles and techniques of transdiagnostic CBT for ED, and aims to normalize eating behavior, reduce the importance given to both weight and shape, and promote adaptive emotional regulation strategies. There is a study in which this app shows positive results in adult ED patients, showing a reduction in overall ED psychopathology [35]. There are other apps targeting ED, but with less empirical support, such as *TCApp* [36, 37], *FFT* [38], *FoodT* [39], or *MT—BD* [40]. All of these applications are aimed at the clinical population, but none of them focus on addressing risk factors before the disorder has developed.

**Fig. 1:**
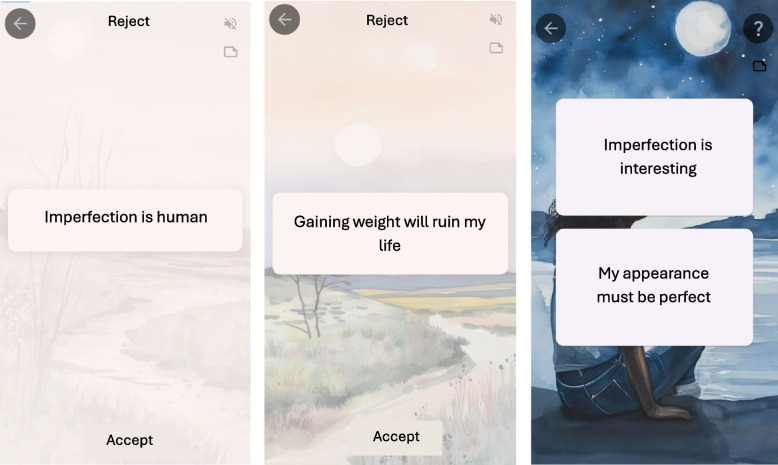
Examples from the GGED-AD application

In Spanish, as far as we know, there is only one mobile application designed to address core beliefs associated with eating disorders. GGED (GG Eating disorders) is a specific module developed in the GGtude platform, an app which is composed of different modules, each of them aimed at working with different mental health problems. GGtude is a commercial application, but users can access the modules under study for free. It is available for Android and iOS, and people can download it by searching for "GG OCD" in the play store or app store. The goal is to provide an easy-to-use tool to work on self-dialogues related to the core beliefs associated with psychological problems in particular. Different studies have supported the efficacy of different modules to reduce the adherence to dysfunctional beliefs associated with obsessive–compulsive disorder [41–43], or with other disorders such as dysmorphic disorder [44, 45] or emotional disorders [46]. The GGED module is composed of a total of 67 levels divided into themes like those worked on in CBT [10]. Some examples of these themes are fear of being judged, perfection of physical appearance, the need to control body weight, self-esteem, fear of food-related thoughts, physical appearance in the media, or fear of shame, among others. The GGED exercises follow the same dynamic as the rest of the modules of the GGtude mobile platform: at each level, statements are randomly presented one by one in the screen in a positive sense (e.g., "An imperfect body is a real body"), and in a negative sense (e.g., "There is nothing worse than getting fat"). (see Fig. 1). The person must identify and “accept” those beliefs that are functional, adaptive, and positive, dragging them to the lower part of the screen; and “reject” those that are dysfunctional, maladaptive, and negative dragging them to the upper part of the screen. The application notifies the user when an error occurs in accepting or rejecting a belief. Otherwise, a message appears on the screen alerting the person that they are accepting a dysfunctional sentence or rejecting a functional one. Thus, exposure to self-affirmations that challenge your maladaptive beliefs can improve your accessibility and ability to generate adaptive self-affirmations. In addition, daily coaching can help users become more aware of their internal dialogue and soften the interpretation of thoughts associated with eating disorders [47].

Preliminary efficacy data with general adult population using GGED for two weeks (3 min per day), showed a decrease in dysfunctional beliefs regarding perfectionism in physical appearance, vulnerability to weight gain, and the importance of thought control, as well as an increase in self-esteem [48].

Considering the potential of the GGED module to modify dysfunctional beliefs associated with eating disorders, as well as the fact that adolescence is a developmental stage in which individuals are vulnerable to the development of these disorders, we adapted the GGED module to the adolescent population (GGED-AD). Thus, the aim of the present study is to describe the design used to evaluate the effectiveness of GGED-AD at decreasing dysfunctional beliefs associated with eating disorders, eating symptoms, and symptoms of depression and anxiety, and increasing body satisfaction and self-esteem among adolescents through a randomized controlled trial. Following the results found in a previous study with GGtude platform for ED in adults [48], we hypothesize that after the use of the GGED-AD app for 14 days in the experimental group, there will be a decrease in the degree of ascription to dysfunctional beliefs associated with ED in adolescents at the primary level; and an increase in body satisfaction and self-esteem and decrease in eating symptomatology at the secondary level. It is also hypothesized that there will be no change in emotional symptoms, as has been found in previous research on the GGED module in the adult population [48], or in this variable with the GGOC module [42, 43]. Results are also expected to be maintained in the subsequent follow-up a month after the use of the app.

## Methods

The present protocol complies with the Standard Protocol Items: Recommendations for Interventional Trials (SPIRIT) checklist [49]. Additional file shows this in more detail (see Additional file 1).

### Sample

The study will include adolescents between the ages of 13 and 16 from an educational center in the Valencian Community.

The inclusion criteria will include (1) ages between 13 and 16, (2) informed consent form signed by both parents or guardians and the adolescents, and (3) access to a smartphone with Internet connectivity.

The sample size was calculated using the free program G*Power (version 3.1.9.7) [50]. To determine the size of the effect of the predictor variables on the response, Cohen’s conventional tables will be used. It was determined that a sample of at least 68 participants achieve a power of a power of 0.805 at alpha = 0.05 and a medium effect size of effect (*f* = 0.15).

### Study design

A randomized controlled trial will be conducted with adolescents who will be randomly assigned by gender to a control or experimental group.

**Fig. 2:**
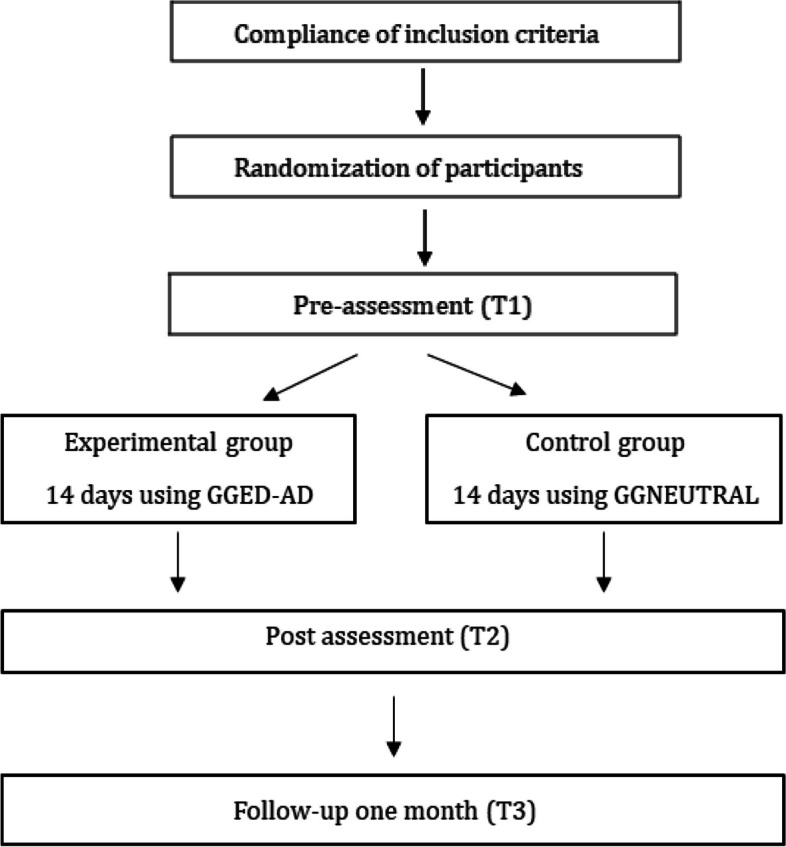
Organizational Chart of the Study. Note. 14 days using GGED-AD: use of the GG Eating Disorders – Adolescents module during 14 days; 14 days of GGNEUTRAL: use of the GG Neutral module during 14 days

The experimental group will perform cognitive training through the GGED-AD module; while the control group will use another module of the app, named GGNEUTRAL (Fig. 2). The study will consist of a pre-assessment (T1) and then participants will have to use the corresponding application for a period of 14 consecutive days, for approximately 5 min a day. After completing all levels, a subsequent evaluation (T2) will be carried out, as well as a follow-up evaluation one month after having completed the app module (T3), in order to evaluate whether the possible changes are maintained over time. Figure 2 and Table 1 show the organizational chart of the study.

**Table 1:**
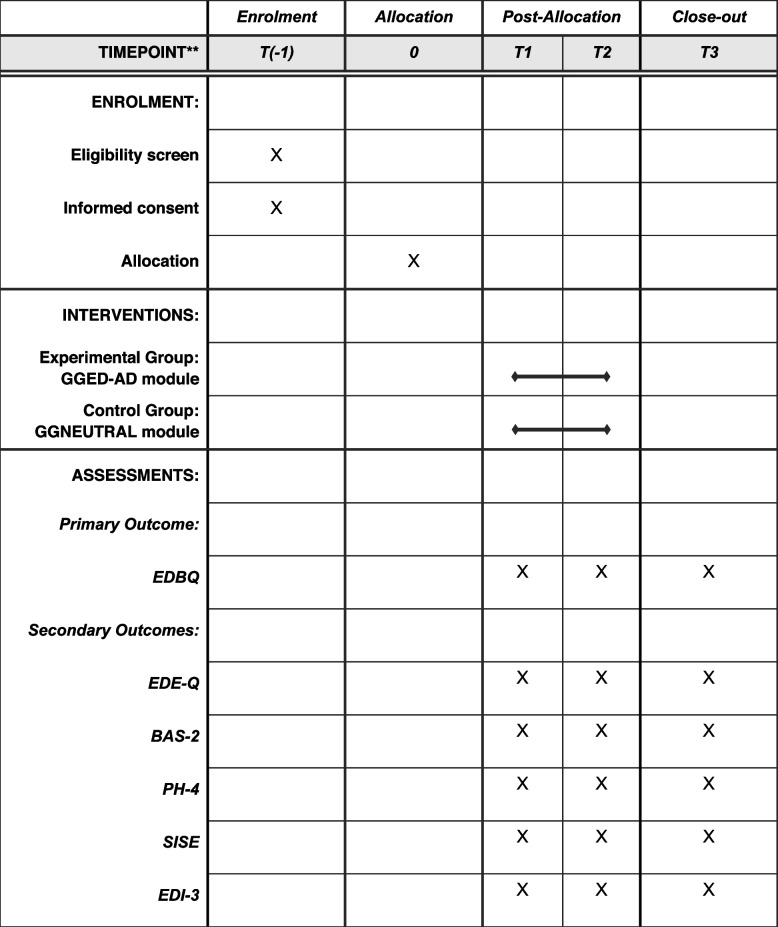
Schedule of enrolment, allocation, intervention, and assessments within according to SPIRIT

Note. T(-1)= enrolment; T0 = allocation; T1= baseline assessment; T2 = post assessment; T3= follow-up assessment 1 month later; EDBQ = Eating Disorder Beliefs Questionnaire; EDE-Q = Eating Disorder Examination Questionnaire; BAS-2 = Body Appreciation Scale-2; PH-4 = The Patient Health Questionnaire; SISE = Single-Item Self-Esteem Scale; EDI-3 = Eating Disorder Inventory

### Intervention

The study groups will complete two different modules of the GGtude platform. The experimental group will complete the GGED module adapted to adolescents (GGED-AD) and the control group will complete the GGneutral module. Both modules are described below.

#### GG eating disorders – Adolescents (GGED-AD)

The GGED-AD is the adaptation of the GGED for adolescents, which is used to work on dysfunctional beliefs associated with eating disorders [51]. The adaptation consisted of three phases. First, a group of researchers with expertise in eating disorders selected statements that may be difficult for teenagers to understand. Of the 708 statements that make up the application, a total of 131 statements were selected. Subsequently, an ad hoc questionnaire was created and administered to adolescents (*n* = 5, *M*_*age*_ = 12, *SD* = 0, 60% female). In this questionnaire, participants indicated their level of understanding of sentences on a visual scale with three response options (green: “I understand it perfectly”; yellow: “I don't quite understand it”; red: “I don’t understand it”). Subsequently, the teenagers had to propose an alternative for the sentences marked as “yellow”. Of the 131 statements selected in the first phase by the group of experts, 88 presented comprehension problems for the participants in this second phase. Finally, in the third phase, the appropriateness of the alternative statements proposed by the participants was evaluated by four psychology PhD researchers (Corberán et al., 2023).

Similar to the GGED module, the GGED-AD is made up of the topics that are normally worked on in CBT in relation to eating disorders [10]. The final version of the GGED-AD includes the following topics: perfect physical appearance, fear of being judged, fear of body fat, body as an object, control of body weight, need for control, fear of complacency, self-esteem, fear of food-related thoughts, physical appearance in the media, fear of shame, negative internal image, and fear of abandonment. As in the adult's version, the sentences from these themes have to be "accepted" or "rejected" based on their adaptiveness. This module will be used by participants in the experimental group.

#### GG neutral module (GGNEUTRAL)

This a module belonging to the GGtude digital platform GGED designed exclusively for research purposes. The operation is similar, sentences appear in the center of the screen, but in this case, the person must accept those sentences that are true (e. g., "Madrid is the capital of Spain"), by dragging them to the top of the screen; and reject those that are false (e. g., "Tokyo is the capital of Spain"), by dragging them to the bottom of the screen. All the statements that compose it are totally independent of the target of the study, thus, there is no sentences related to weight or physical appearance. To use this module in the present study and facilitate its understanding, some sentences were modified to the Spanish context of the teenagers. For example, “Arizona is a state in the USA” was replaced by “Valencia is a city in Spain”. This module will be used by participants in the control group.

### Outcome measures

#### Primary outcomes

*Eating Disorder Beliefs Questionnaire (EDBQ;* Cooper et al., 1997 [52]). It is composed of 32 items that examine core beliefs about weight, physical appearance and eating that are associated with eating disorders. Items are rated on an analog scale from 0 to 100, being 0 “I do not usually believe this at all” and 100 “I am usually completely convinced that this is true”. It contains four subscales: negative self-beliefs, weight and shape as a means of acceptance by others, weight and shape as a means of self-acceptance, and control over eating. In its original version, it presents Cronbach's α values between 0.94, 0.95, 0.90 and 0.89 respectively. Given that there is no validated Spanish version of this questionnaire, the present study will use the translation-retranslation method [53]. First, a minimum of two native Spanish-speaking members of the research team will perform the forward translation of the original questionnaire into Spanish independently. Subsequently, a meeting will be held with the individuals who carried out the forward translation and the rest of the research team. During this meeting, a consensus on the translation of the questionnaire will be reached. This Spanish version will be sent to a native English speaker who, totally blind to the original version, will back-translate it into English. Again, the research team, composed by mental health professionals as well as psychometricians, will check that the back-translation and the original questionnaire match. In the present study, the psychometric properties of the instrument translated into Spanish will be calculated to check its reliability and validity.

#### Secondary outcomes

*Eating Disorder Examination Questionnaire* (*EDE-Q*; Fairburn & Cooper, 1993 [54]; Spanish version: Peláez-Fernández et al., 2012 [55]). It is a 36-item self-report questionnaire that assesses the range, frequency and severity of behaviors associated with a diagnosis of an eating disorder. It comprises four subscales (restriction, food concern, weight concern and figure concern) which are composed of attitudinal and behavioral items. For the present study, we will only use 22 attitudinal items. Items are scored on a 7-point Likert-type scale, ranging from 0 (“No day/ No time/ Not at all”) to 6 (“Every day/ Always/ Completely”). Its original version and its validation in Spanish present an adequate internal consistency (Cronbach's α is 0.86, 0.75, 0.93 and 0.74 for the four scales).

*Body Appreciation Scale-2 (BAS-2;* Tylka & Wood-Barcalow, 2015 [56]; Spanish version: Swami et al., 2017 [57]). It is a single-factor questionnaire that evaluates body satisfaction with 10 items on a 5-point Likert scale (from 1 = “Never” to 5 = “Always”). Both its original and Spanish versions present high internal consistency with Cronbach's α above 0.90.

*The Patient Health Questionnaire (PHQ-4;* Kroenke et al., 2009 [58]; Spanish version: Lenz & Li, 2022 [59]). This is a 4-item measure of two factors: depression and anxiety. It assesses the symptoms experienced by participants during the 2-week period before taking the survey (two items are used for the main symptoms of anxiety and two for depression). The response format is a 4-point Likert-type scale (from 0 = “Never” to 3 = “Almost every day”), and its validation presents adequate psychometric qualities, with an internal consistency (Cronbach's α) of 0.86 (depression) and 0.91 (anxiety).

The *Single-Item Self-Esteem Scale (SISE*; Robins et al., 2001 [60]) is a self-report measure in which the person has to indicate how the statement “I have high self-esteem” describes him/her on a 9-point scale (from 1 = "Not very true for me" to 9 = "Very true for me"). This scale presents high test–retest reliability and a similar criterion validity coefficient with the Rosenberg Self-Esteem Scale (Rosenberg, 1965 [61]) above 0.80.

*Eating Disorder Inventory (EDI-3;* Garner, 2004 [62]; Spanish version: Elosua et al., 2010 [63]). It evaluates 18 scales with a total of 91 items, with a 6-point Likert scale, ranging from 0 = “Never” to 5 = “Always”. However, in this study only the interpersonal insecurity (7 items) and perfectionism (6 items) factors will be used. These factors present Cronbach's α values of 0.93 and 0.75 respectively at its Spanish validation [63].

### Study procedure

First, an interview will be held with the school leadership team and/or heads of the guidance department of different schools in the Valencian Community, to explain the research objectives, describe the evaluation instruments, request permission to apply them, and encourage their collaboration. The schools interested in participating will distribute an information sheet specifying the objectives and characteristics of the study among the students and their families. Parents of students interested in participating in the study will have to sign the informed consent form authorizing their children to participate in the research. Students themselves must also sign it. The participants that enroll in the study will be randomly assigned by gender to the experimental and control groups using the Random.org program. On the first day of the intervention at the school center, there will be an informative session for the students to explain guidelines for their participation in the study. This will include the importance of being quiet and the option to stop participating at any time. Additionally, there will be assistance provided for the administration of the first evaluation (T1). T1 evaluation will include the evaluation instruments, and sociodemographic data, which will include information on gender, age, school, grade and academic group, height, weight. Once T1 is completed, participants will download the corresponding app to start completing the daily levels. The experimental group will use the GGED-AD app, and the control group will use the GGNEUTRAL app, named GGA and GGN respectively to avoid giving excessive information to participants. Specifically, both apps are divided into 14 blocks, and the participants will do one block each day. Each block has 4–5 levels, which is equivalent to approximately 4–5 min. During this time, members of the research group will be present in the classroom on school days, to solve possible doubts and to control the use of the application. During non-school days participants will have to complete the app at home. After completing the app, all participants will carry out the second evaluation (T2). This evaluation will be composed of the same set of questionnaires completed at T1. Once T2 is completed, the adolescents will receive instructions to not use the application until we contact them again to conduct the follow-up after one month (T3) to evaluate if the possible changes achieved are maintained over time. If someone has difficulties or feels uncomfortable during the completion of the evaluation of the module, they will be free to speak to members of the research group who, in case of it being necessary, will be referred to the school counsellor, and in addition will show them different services where they could find help for possible mental health problems.

### Blinding

Adolescents will be randomly assigned to either the intervention or control group and will not be informed about which group they are in. However, in this scenario, maintaining the blinding is not possible because participants are aware of the two different apps. Each individual will be checked to determine whether they use the GGED-AD or GGNEUTRAL app. Research team members need to be aware of which group each participant belongs to in order to guide them correctly through the application process.

### Statistical analysis

Statistical analyses will be performed using the free software R. To avoid overly optimistic estimates of the efficacy of the experiment, an intention to treat (ITT) approach will be used, using the method of the multivariate imputation by chained equations algorithm (MICE) [64].

Descriptive statistics will be used to report means, standard deviations and frequencies. In addition, tests will be performed to evaluate differences between the different variables considered in the study. To predict the different response variables under study, mixed models for repeated measures (MMRM) will be used, in which the outcomes measures are dependant variables, and the independent variables are group of the study (experimental or control), gender (male/female/other) and time (T1, T2, and T3). In addition, the baseline scores for each of the factors evaluated will be used as covariates to control that the T1 scores do not affect the results of the efficacy of the app.

### Data management and data monitoring

All self-report questionnaires will be completed by participants online via Lime Survey, a secure server of the University of Valencia. This server meets the security requirements of the GDPR. Participants will use a unique personal identification number (ID), so the data storage will be anonymous. The ID will be used to match the data. In addition, two control questions (e.g., “Please, answer “always” in this question”) are included throughout the evaluations to try to avoid random answers.

## Results

This study was approved by the Ethics Committee of Research in Humans of the University of Valencia on March 14, 2023, with register code 2,557,724; and accepted and published in ClinicalTrials.gov, with ID: NCT06039514. The planned data collection period is one month, and a follow-up one month later. In addition, two weeks prior to the data collection period, parents and/or guardians will be contacted to deliver the informed consent forms and collect them signed.

## Discussion

### Expected outcomes

With respect to the primary outcomes, a decrease in the degree of ascription to dysfunctional beliefs associated with eating disorders is expected in the experimental group at the T2 assessment. These changes are also expected to be maintained in the follow-up evaluation (T3) in this group. No changes are expected in the control group. The cognitive training on the adaptive and maladaptive beliefs related to ED is the principal objective of the app. Therefore, a reduction in ED dysfunctional beliefs is expected in the participants of the experimental group after the use of the GGED module, given that in other studies conducted with different modules of the GGtude platform this change was also found in the thoughts worked on (e.g., obsessive-compulsive disorder [41–43], body image [44, 45, 65], anxiety [47, 66, 67], emotional disorders [46, 68], and self-esteem [69]). Moreover, the preliminary efficacy data of GGED used in the adult population also found these changes at post-intervention [48]. It is expected that those results will be replicated in adolescents.

Regarding secondary outcomes, an increase in body satisfaction and self-esteem, and a decrease in eating symptomatology are expected in the experimental group at T2. Their maintenance is also expected at T3. None of them are expected in the control group. Changes in self-esteem and in diverse symptomatology have been found in other studies with other modules, such as obsessive–compulsive disorder [41–43] or emotional disorders [46]. With respect to body satisfaction, there are positive results [44, 45] with another module of the GGtude platform targeting body image concerns in young people (between 20 and 30 years of age). Some maladaptive beliefs worked on in this module (e.g., inflated importance of one’s appearance, perceiving the body as an object, and appearance related perfectionism) are also included in the GGED-AD module. In the same way, these results are also expected in the present study which is aimed at ED in the adolescent population. As for emotional symptomatology, no changes are expected, given that the studies with other modules have not found changes [42, 42, 48], or these were not maintained over time [65].

### Expected contribution

The GGED-AD application may be a step forward in the prevention of eating disorders in adolescents, who are at a period of greater vulnerability in their development [5, 70]. Although different adolescent eating disorder prevention programs exist, this app provides a different tool to address risk factors, given that none of the prevention programs that have been evaluated on their effectiveness are based on a mobile app. It is the first mobile application on eating disorders in Spanish based on cognitive behavioral therapy. It helps to do deeper cognitive work on the dysfunctional beliefs that develop and/or maintain these disorders. In addition, it is easy to access and use, which makes it easier to reach more people. Moreover, in the school setting, GGED will be easy to implement. A simple explanation to teachers will enable them to help and oversee its use, enhancing adherence, which is the main difficulty in the field of mobile application interventions [71, 72].

### Challenges

Some challenges that may be encountered when carrying out the study are: (1) making a schedule that interrupts the lessons as little as possible; (2) that the participants use the application properly, in silence and focused while reading the sentences; (3) that participants complete the app during weekends. In order to overcome these challenges, the researchers will meet with the team of teachers to agree on a procedure that will cause as little disruption as possible to their work. In addition, the researchers will be there every day to accompany the adolescents as they complete the app, to remind them of the importance of completing the app seriously, to see that they do not answer randomly and to encourage them to complete the app over the weekend. It is possible that the number of dropouts will be higher than expected. Thus, the goal is to motivate participants as much as possible to minimize the dropout rate.

### Supplementary Information

Supplementary Material 1.

## Data Availability

No datasets were generated or analysed during the current study

## Abbreviations

ED: Eating disorder
GGED-AD: GG Eating disorder – Adolescents
CBT: Cognitive-behavioral therapy
ID: Identification number
GGED: GG Eating disorders
EDBQ: Eating Disorder Beliefs Questionnaire
EDE-Q: Eating Disorder Examination Questionnaire
BAS-2: Body Appreciation Scale-2
PH-4: The Patient Health Questionnaire
SISE: Single-Item Self-Esteem Scale
EDI-3: Eating Disorder Inventory
